# Temporal changes in diet and mortuary practices among the Jomon people based on human skeletal remains excavated from the Kawaji shell mound in Aichi Prefecture, Japan

**DOI:** 10.1537/ase.250522

**Published:** 2025-07-17

**Authors:** Soichiro Kusaka, Yasuhiro Yamada

**Affiliations:** 1 School of Humanities, Tokai University, Shizuoka, 3-20-1 Orido, Shimizu-ku, Shizuoka City, Shizuoka, 424-8610, Japan; 2 Graduate School of Humanities, Tokyo Metropolitan University, Tokyo, 1-1 Minami-Osawa, Hachioji City, Tokyo, 192-0397, Japan

**Keywords:** Jomon period, hunter-gatherers, carbon, nitrogen, stable isotope

## Abstract

People of the Jomon period in Japan led a hunter-gatherer lifestyle and actively engaged in fishing in coastal areas. On the Atsumi Peninsula, which is located in the southern part of Aichi Prefecture, a number of shell mounds from the Jomon period have been located. More than 30 human skeletal remains have been excavated from the Kawaji shell mound, located at the tip of the Atsumi Peninsula. The Kawaji shell mound was formed from the Middle to Final Jomon period and is thought to be mainly from the Late Jomon period. This study aimed to investigate temporal changes in diet and their relationships with subsistence activities and mortuary practices, which include types of ritual tooth ablation and the location pattern of burials within the site, during the Jomon period. The study material consisted of 20 human skeletal samples from the Kawaji shell mound. Diet was inferred using carbon and nitrogen isotope analysis of bone collagen, and age by radiocarbon dating. Age showed values from the late Middle to the Final Jomon and the Initial to Middle Yayoi periods. Dietary dependence on marine resources increased during the Late Jomon period, and then decreased in the Initial to Middle Yayoi periods. Transition patterns in ritual tooth ablation were also found. Burial subgroups within the shell mound were recognized in each phase. The findings from this case study help shed light on the temporal changes that occurred in the subsistence activities and mortuary practices of the Jomon people.

## 1. Introduction

During the Jomon period (15540–2450 calBP), people living in the coastal areas led a hunter-gatherer-fisher lifestyle (Akazawa, 1999). They made Jomon pottery and lived in sedentary pit dwellings. Their staple diet consisted primarily of nuts (e.g. chestnuts, acorns), and they hunted mammals (e.g. deer, wild boar) and caught a variety of fish and shellfish. Dietary reconstruction can be conducted through carbon and nitrogen stable isotope analysis of bone collagen (Minagawa and Akazawa, 1992). Carbon and nitrogen isotope ratios of animals are enriched ascending through the trophic levels (Minagawa and Wada, 1984; Post, 2002). A large difference in isotope ratios between terrestrial and marine resources allows the dietary dependence on marine resources to be assessed (Chisholm et al., 1992). Stable isotope analysis has been used to examine geographical variations in diet among human skeletal remains from the Jomon period (Minagawa and Akazawa, 1992; Yoneda et al., 2004). Intra-site variation in diet has also been evaluated at several sites along the coast of Honshu (Kusaka et al., 2010). Temporal changes in diet have been reported at some sites (Kusaka et al., 2015, 2018). Given that age and dietary habits can be studied, it is also considered possible to explore how hunter-gatherers adapted to climatic and environmental changes during the Jomon period.

**Figure 1.**
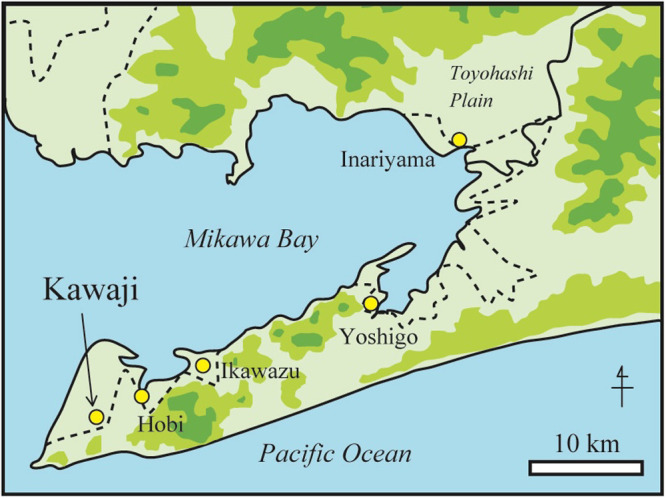
Map of the Kawaji shell mound.

The Atsumi Peninsula in Aichi Prefecture, Japan, is the location of numerous shell mounds from the Jomon period (Yamada, 2022a, 2022b). About 30 human skeletal remains, mainly from the Late Jomon period, have been discovered in the Kawaji shell mound, which is located at the western end of the Atsumi Peninsula (Harada et al., 1995; Figure 1). The Kawaji shell mound has also been referred to as the Kameyama shell mound, but Kawaji is used in this paper. The Hobi shell mound is located 4.5 km northeast of the Kawaji shell mound, and further east are the Ikawazu and Yoshigo shell mounds, which have each yielded more than 100 human skeletal remains. Bioarchaeological analyses, such as bone metrics, ancient DNA, and strontium isotopes, have been applied to these materials (Kondo et al., 2022; Kusaka et al., 2022; Mizushima et al., 2022; Waku et al., 2022; Yamada, 2022b). Thus, the Mikawa Bay coast is characterized by a large number of shell mounds from which Jomon skeletal remains have been excavated, thereby making it possible to examine how changes in the climate and environment affected the lives of the people of the Jomon period.

Chronology based on radiocarbon dates of Jomon pottery is used to fit the age of human skeletal remains to time divisions of the Jomon and Yayoi periods (Table 1). Regarding the ages of the Jomon pottery types, the Incipient Jomon period is 15540–11345 calBP, the Initial Jomon period is 11345–7050 calBP, the Early Jomon period is 7050–5415 calBP, the Middle Jomon period is 5415–4490 calBP, and the Late Jomon period is 4490–3220 calBP (Kobayashi, 2017). Based on the ages of the Jomon and Yayoi pottery in Aichi Prefecture, the first half of the Final Jomon period is 3220–2850 calBP (Yamamoto, 2008). The Initial Yayoi period is 2850–2450 calBP, and the Early Yayoi period is about 2460–2305 calBP (Yamamoto, 2008; Kobayashi, 2017). The Initial Yayoi period corresponds to the second half of the Final Jomon period (Shitara and Kobayashi, 2004). This means that the Initial Yayoi period is the transitional time from the Jomon to Yayoi cultures. The Middle Yayoi period is about 2350–2100 calBP (Fujio and Ozaki, 2009). Even in the Middle Yayoi period, Jomon culture continued in the people of the Jomon shell mounds around Mikawa Bay (Kusaka et al., 2018). For the purposes of this study, we simply use the terms Final Jomon and Initial Yayoi for the first and second half of the Final Jomon period, respectively.

Sea levels in the Tokai region have been reconstructed by investigating the Mazukari shell mound in Aichi Prefecture. This shell mound, located at a depth of 10 m below ground level, dates to the Early Jomon period (Maeda et al., 1983). At 9900–8540 calBP (8590 ± 260 BP), the sea level, which was –13 m, rose rapidly and reached about +4.5 m by 7000 calBP. Subsequently, the sea level fell, reaching about +1 m by 5270–4350 calBP (4560 ± 170 BP), and then rose to a height of +2 m by 3370–2740 calBP (3220 ± 140 BP). It is therefore considered that a ‘Yayoi minor sea level retreat’ occurred between the Final Jomon and Early Yayoi periods, when the sea level dropped to –1 m. These changes in sea level would likely have affected fishing activities in the vicinity of the Kawaji shell mound.

Climate change during the Holocene has been reconstructed by analyzing diatom assemblages in seafloor cores (Koizumi, 2008). An analysis of seafloor cores from offshore Kashima, Ibaraki Prefecture, where the Kuroshio Current flows, found that the climate was about 1–2°C warmer in the middle Holocene (8200–3300 calBP). Subsequently, a gradually colder climate has been reconstructed. According to a reconstruction of seawater surface temperatures based on sediment cores from Hiroshima Bay, cold periods occurred between 2950–2890 calBP and 2720–2570 calBP (Kawahata et al., 2017). These findings raised the question of how such climatic changes could have affected the diet of the Jomon people.

To answer this question, the age of human skeletal remains needs to be estimated by radiocarbon dating of bone collagen. Radiocarbon dating of human skeletal remains from the Ikawazu shell mound in Aichi Prefecture revealed that the remains were from the Final Jomon to Initial Yayoi periods (Kusaka et al., 2015). In addition, dating of human skeletal remains from the Inariyama shell mound, located east of Mikawa Bay, found them to be from the Late Jomon to Middle Yayoi period, with an increasing dependence on marine products seen with younger age (Kusaka et al., 2018). Ritual tooth extraction has been observed in human bones from this period. The extraction of four mandibular incisors is termed type 4I, while that of two mandibular canines is termed type 2C (Harunari, 1979, 2002). The extraction method for four mandibular incisors and two canines is referred to as type 4I2C. Among the remains from Inariyama, the tooth extraction type changed from type 4I to type 2C in males and from type 4I to type 4I2C in females. Such chronological changes in diet and the type of tooth extraction may also be observed at other sites.

The Kawaji shell mound is located on a medium terrace surface known as the Fukue-men, with an alluvial lowland to the northwest. Based on excavated artifacts, the main chronology of the Kawaji shell mound is the Middle, early half of the Late, and end of the Final Jomon period. It is estimated that tidal flats developed in the immediate vicinity of the site during the Middle Jomon period, then moved away from the coast with the subsequent retreat of the sea, and then again during the Late Jomon period (Harada et al., 1995). These changes in sea level and the coastal environment may have affected the diet of the Jomon people.

Given this background, to deepen our understanding of the adaptations made by the Jomon people in response to changes in their environment and culture, the present study aimed to clarify temporal changes in the diet of people of the Jomon period by examining the chronology of human skeletal remains excavated from the Kawaji shell mound and diet indices based on carbon and nitrogen stable isotope ratios, as well as the temporal relationships with changes in sea level and climate and the tooth extraction practices of human skeletal remains and burial locations within the shell mound.

## 2. Materials and methods

### 2.1 Kawaji shell mound

The Kawaji shell mound is located in the western part of the Atsumi Peninsula, in Tahara city, Aichi Prefecture. In 1922, it was excavated by Dr Kiyono, who found 23 human skeletal remains (Kiyono, 1969). In the present study, 22 of the 23 specimens were analyzed, nine of which have been previously reported in regard to their carbon and nitrogen isotope ratios by Kusaka et al. (2010), and samples were newly collected from 13 specimens (Table 2). The sex and age at death for new samples were estimated using the same procedure as that reported in Kusaka et al. (2010). Identification of ritual tooth ablation is based on the observation of the dentition: missing teeth with partial or full alveolar resorption were recorded as extracted teeth. We followed the tooth ablation types (2C and 4I2C) classified by Harunari (1979).

Most human skeletal remains were excavated from the lower part of the shell layer, with the cephalic direction in many cases being from east to northeast. Overall, many broken bones were seen, and the practice of tooth extraction was also observed in several specimens. The shell layer is 45 cm thick at the thickest point, and high proportions of Japanese littleneck clams, Asiatic hard clams, and oysters were reported (Kiyono, 1969). Stone weights used for net fishing were abundant among the stone tools. Most of the pottery is parallel to the Horinouchi style of the early Late Jomon period (Harada et al., 1995). Osteological studies of human bones excavated from the Kawaji shell mound have been reported (Yorimitsu, 1935). In 1991, the Kawaji Site Research Group, commissioned by the Atsumi Town Board of Education, detected eight Late Jomon dwelling sites and found two human skeletal remains (Mouri and Kinoshita, 1993). This survey reports on not only pottery from the early part of the Late Jomon period, but also many objects from the later part of the Late Jomon period.

In 1993, a human bone was found in a grave during excavation by the Aichi Prefecture Center for Archaeological and Cultural Heritage in association with road construction (Harada et al., 1995). This individual was reported to be a short female (144 cm tall) with robust lower limb bones. The pottery excavated during this survey was classified into three periods: the early to late Middle Jomon, Late Jomon, and end of the Final Jomon (Harada et al., 1995). The pottery from the end of the Final Jomon period included Kashio-style pottery and jars from the Middle Yayoi period, indicating that the end of the Final Jomon period was a transitional time from Jomon to Yayoi culture, although no evidence of rice agriculture from the Yayoi period was found in this shell mound. The shellfish excavated in this survey included many marine intertidal sand snails and clams, which live in the shallow waters of the inner bay. Among fish species, black sea breams and sea bass from the inner bay were found, as well as red sea bream, sharks, and tuna from the open sea. It is assumed that these people made extensive use of the marine resources of the open sea (Harada et al., 1995). Mammals, mainly Japanese deer and wild boar, have also been excavated. The stone tools discovered, mainly consisting of stone net sinkers of the Late Jomon period, suggest that the fishing activities in coastal areas were active, a trend that differs from the examples from the Ikawazu and Yoshigo shell mounds, where a high proportion of stone arrowheads were found (Harada et al., 1995).

In 2018, the Tahara City Board of Education carried out a survey to ascertain the condition of the site. The elevation of the ground surface was approximately 4.2 m, and that of the ground floor was 3.1–3.4 m. Confirmation of the distribution of shell layers and artifacts pointed out the possibility of a discrepancy between the locations of human graves excavated in the Kiyono report and the actual extent of the main part of the shell mound (Masuyama et al., 2024).

### 2.2 Collagen extraction and stable isotope measurements

Collagen extraction from bone samples was also carried out in the laboratory at Tokai University. The bones were weighed and placed in NaOH (0.2 M solution) overnight, and then rinsed with purified water and lyophilized. Next, they were ground with a hammer and mill, placed in cellulose tubes, and demineralized in HCl (1 M solution) before being returned to neutral and centrifuged. The precipitate was then heated at 95°C overnight. Finally, the samples were filtered and lyophilized to yield gelatinized collagen.

Carbon and nitrogen isotope ratios were then measured using a mass spectrometer equipped with an elemental analyzer (EA-IRMS; Thermo Fisher Scientific, Inc., Waltham, MA, USA) at the Research Institute for Humanity and Nature in Kyoto, Japan. Carbon and nitrogen isotope ratios were expressed by the delta notation as follows:

δ^13^C = (R_sample_/R_standard_ – 1) × 1000 (‰)

where R means ^13^C/^12^C for δ^13^C and ^15^N/^14^N for δ^15^N. The δ^13^C and δ^15^N values were reported against the Vienna Peedee Belemnite standard and atmospheric nitrogen, respectively. The measurement error was <0.2‰ for both δ^13^C and δ^15^N.

Graphitization of bone collagen and radiocarbon dating using an accelerator mass spectrometer (compact AMS, NEC 1.5SDH) were carried out by Paleo Labo Co., Ltd. (Kobayashi et al., 2007). Calibrated ages were calculated using the OxCal 4.4 program (Bronk Ramsey, 2009) with the IntCal20 and Marine20 datasets (Heaton et al., 2020; Reimer et al., 2020). Calibration curves were mixed according to the marine food dependence of each sample, calculated using the δ^13^C of collagen, that of terrestrial mammals plus 1‰ isotopic fractionation (–20.7‰), and that of marine fish bones plus 1‰ (–9.6‰; Kiriyama and Kusaka, 2017). Regional corrections for the marine reservoir effect were considered for the latest calibration curve (–69 ± 36; Shishikura et al., 2007).

Statistical analysis of carbon and nitrogen isotope ratios was performed using JMP 18 (SAS Institute Inc., Cary, NC, USA), with the level of statistical significance set at 0.05.

## 3. Results

The results of collagen extraction from the human skeletal remains excavated from the Kawaji shell mound showed C/N ratios ranging from 3.3 to 3.9 (Table 2). The C/N ratios of well-preserved collagen should be between 2.9 and 3.6 (DeNiro, 1985); 20 samples were within this range. Therefore, it was assumed that the collagen from these samples was of biological origin based on the stable isotope ratios. Two individuals (Nos. 4 and 20) were excluded from the analysis because their collagen had high C/N ratios and was poorly preserved.

**Figure 2.**
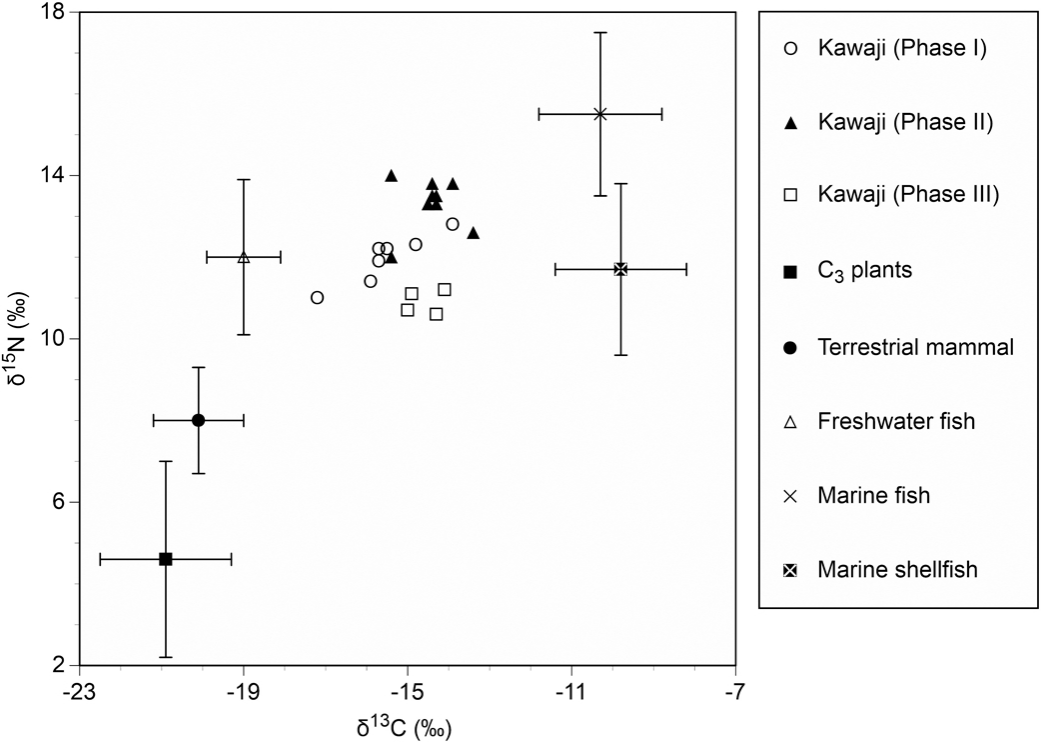
The δ^13^C and δ^15^N values of Kawaji human skeletal remains and food resources. The mean δ^13^C and δ^15^N values of food resources with 4.5‰ and 3.4‰ added as isotopic fractionations, respectively, were cited from Kiriyama and Kusaka (2017), Kusaka et al. (2010), and Yoneda et al. (2004).

The mean δ^13^C value of the Kawaji individuals was –14.9‰ ± 0.9‰ (range, –17.2‰ to –13.4‰) (Table 3, Figure 2). The mean δ^15^N value was 12.4‰ ± 1.1‰ and ranged from 10.6‰ to 14.0‰. The dietary dependence on marine resources of each individual was calculated using a linear mixing model of the δ^13^C values of bone collagen. The mean dietary dependence on marine food was 52.8% ± 8.0% (range, 31.8–66.2%).

**Figure 3.**
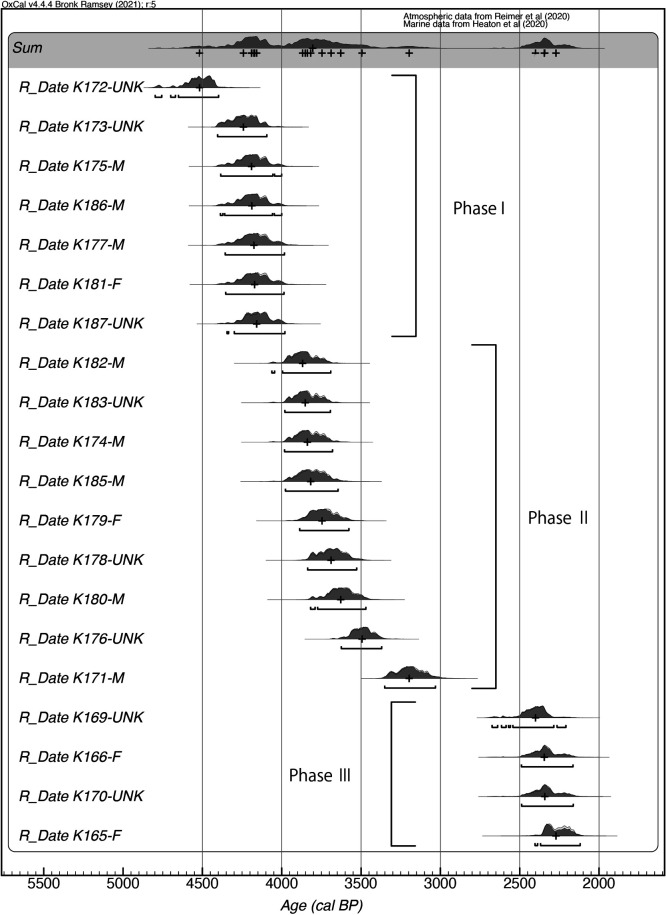
Radiocarbon dates of Kawaji human skeletal remains.

The results of radiocarbon dating on bone collagen yielded dates of 4170–2470 BP (Table 2). The calibration curves of IntCal20 and Marine20 were mixed depending on the marine food dependence of each individual. The calibration of radiocarbon dates revealed a median ranging from 4520 to 2270 calBP (Figure 3). Regarding the 2σ probabilities of the Kawaji skeletal remains, one individual was classified as the end of the Middle and Late Jomon period, fourteen as the Late Jomon period, one as the Late and Final Jomon period, three as the Initial to Middle Yayoi period, and one as the Early and Middle Yayoi period (Table 2).

As the summed probability plot presents three peaks, three phases were set up for categorical comparison of the isotopic data: Phase I, the end of the Middle Jomon and the first half of the Late Jomon period (*n* = 7 individuals); Phase II, the second half of the Late Jomon and Final Jomon period (*n* = 9 individuals); and Phase III, the Initial, Early, and Middle Yayoi period (*n* = 4 individuals; Figure 3). One Middle and Late Jomon individual (No. 8) and one Late and Final Jomon individual (No. 7) may be considered outliers in each phase. However, they were not excluded because their archaeological condition indicated they were contemporaneous with other individuals. The δ^13^C values for bone collagen were not significantly different among these three phases (Table 3; Kruskal–Wallis test, *χ*^2^ = 5.7, *P* = 0.057). The same tendency for the statistics of the δ^13^C values was observed when one Middle and Late Jomon individual (No. 8) and one Late and Final Jomon individual (No. 7) were excluded (Kruskal–Wallis test, *χ*^2^ = 4.2, *P* = 0.125). The δ^15^N values were significantly different between the three phases (Kruskal–Wallis test, *χ*^2^ = 13.6, *P* < 0.01). The δ^15^N value was higher for Phase II than for Phases I and III, but did not differ between Phases I and III (Steel–Dwass test; Phases I and II and Phases II and III, *P* < 0.05; Phases I and III, *P* > 0.05). Significant differences in δ^15^N values were found among the three phases, except for the above two individuals (Kruskal–Wallis test, *χ*^2^ = 12.6, *P* < 0.01). The δ^15^N values of three phases differed from each other (Steel–Dwass test, *P* < 0.05 for the three pairs).

**Figure 4.**
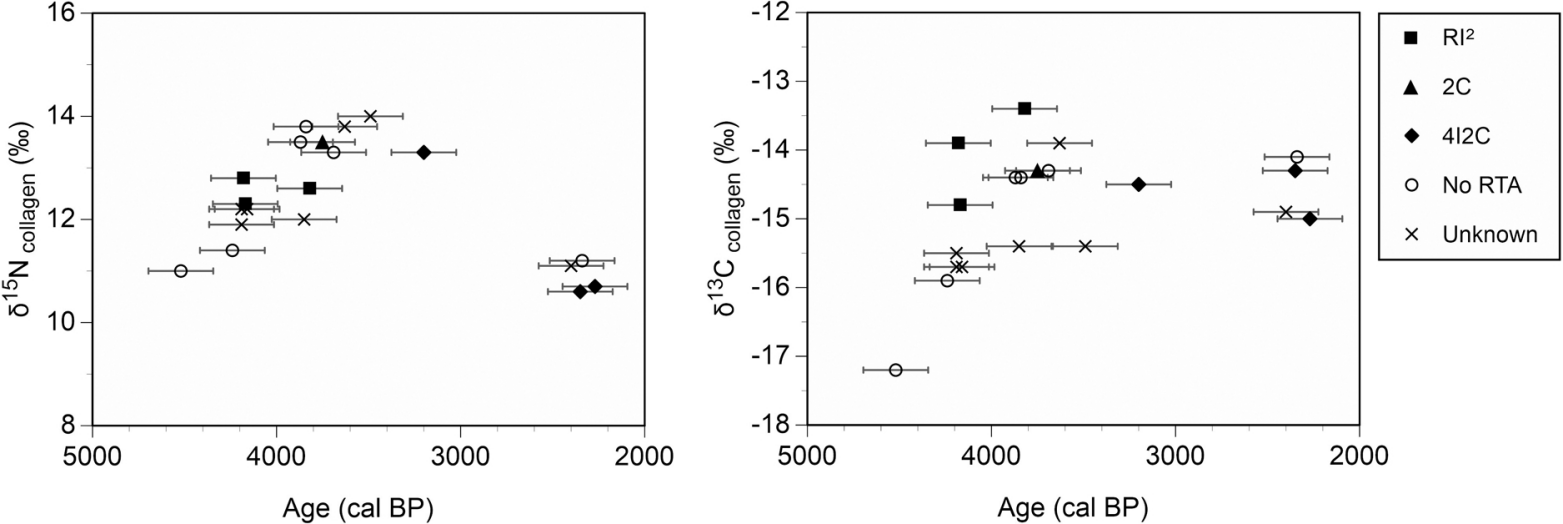
The δ^13^C and δ^15^N values and radiocarbon dates of Kawaji human skeletal remains divided by ritual tooth ablation types. The average 2σ range of calibrated dates was 351 years, and the error bars represent half the average, ±176 years.

The types of ritual tooth ablation differed according to the phase (Figure 4). Individuals of Phase I displayed evidence of extraction of the maxillary and mandibular lateral incisors. Individuals of Phase II displayed extraction of the maxillary lateral incisor and canine, and one type 2C. The individuals in Phases II and III included type 4I2C individuals.

**Figure 5.**
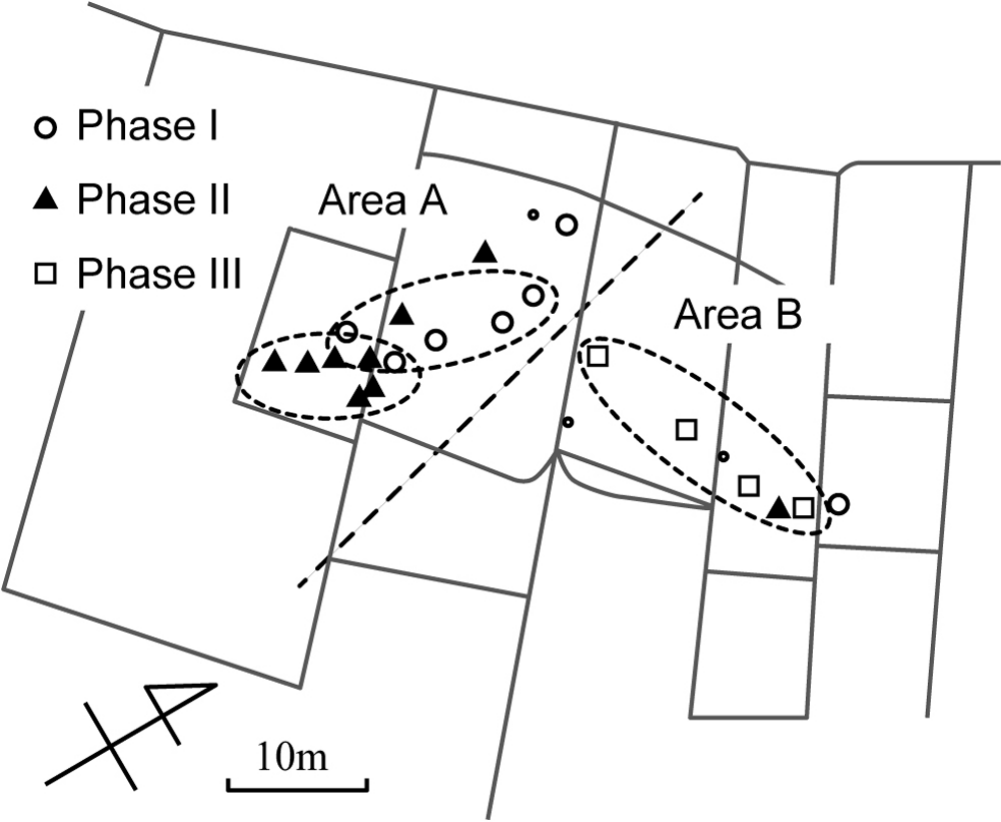
Locations of burial sites in the Kawaji shell mound. Burial subgroups in each phase are indicated in the dashed circles.

The burial location in the shell mound has also been reported (Kiyono, 1969). Many individuals were buried in the western part of the shell mound, with a smaller number buried in the eastern part (Figure 5). Most of the Phase I and II individuals were buried in the western part of the shell mound, whereas the Phase III individuals were buried in the eastern part.

## 4. Discussion

The results of radiocarbon dating of human skeletal remains excavated from the Kawaji shell mound yielded a median date range of 4520–2270 calBP. Sixteen individuals were classified as belonging to the end of the Middle to Late and Final Jomon period, and four to the Initial to Middle Yayoi period. The Kawaji shell mound was considered to belong to the Middle, Late, and end of the Final Jomon period based on excavated pottery, which was abundant for the Late Jomon period (Harada et al., 1995). This finding is consistent with the fact that many of the individuals belonged to the Late Jomon period. However, only one and four individuals were identified from the Middle Jomon and Early and Middle Yayoi periods, respectively. Some shell layer locations had been destroyed before the excavation (Kiyono, 1969). Thus, the small number of human skeletal remains of the Middle Jomon and Initial to Middle Yayoi periods might have been the result of excavation bias.

The temporal difference between the δ^13^C and δ^15^N values revealed that the δ^15^N values were lower for Phase I than for Phase II, which indicates that dietary dependence on marine resources was lower during the end of the Middle Jomon and early half of the Late Jomon period. The δ^13^C and δ^15^N values of food resources are higher with increasing trophic level, and the δ^13^C and δ^15^N values of bone collagen have been used to examine dietary dependence on terrestrial and marine resources, as they differ substantially between terrestrial and marine resources (Minagawa and Akazawa, 1992; Minagawa, 2001; Kusaka et al., 2010). The plot of the δ^13^C and δ^15^N values for Phase I shows a positive correlation, suggesting that the dietary differences between individuals resulted from the incorporation of different proportions of marine resources. The dietary sources for high δ^13^C and δ^15^N values of individuals would be high rather than low trophic-level fish and shellfish. The δ^13^C and δ^15^N values for Phase II indicate a high intake of high trophic-level marine fish.

The δ^15^N values of individuals in Phase II were higher than those of individuals in Phase III. The proportion of stone net sinkers increased during the Late Jomon period, suggesting active net fishing was happening along the coast (Harada et al., 1995). The analysis of animal skeletal remains revealed bones from fish that lived in the open sea (Harada et al., 1995). The results of the analysis of animal skeletal remains from the shell mounds on the Atsumi Peninsula suggest that fishing in the open sea was happening at the western sites (Toizumi, 2008). The increasing δ^15^N values for Phase I and higher values for Phase II compared with Phase III are in accordance with these inferences.

The paleoclimate during the Late Jomon period was warm, with fluctuations of 2–3°C, and cooler temperatures during the Final Jomon to Early Yayoi period were suggested (Koizumi, 2008). The sea level rose up to +2 m during the Late Jomon period, and fell throughout the Final Jomon to Yayoi period. The sea level was lower in the early half of the Late Jomon period, and the coastline was far from the Kawaji shell mound (Harada et al., 1995). The sea level rose in the latter half of the Late Jomon period, and tidal flats developed near the shell mound. Such changes in the paleoenvironment might have enhanced the fishing activities along the coast and in the open sea during the latter half of the Late Jomon period.

The δ^15^N value of individuals during the Initial to Middle Yayoi period was low, which indicates a lower proportion of marine fish and shellfish. Despite the period, no remains or artifacts of rice agriculture were excavated. These individuals would have been hunter-gatherers by tradition from the Jomon period. The relatively cool climate and coastline located far from the site might have reduced fishing activities.

The extraction pattern regarding ritual tooth ablation appears to have changed over time (Figure 4). The maxillary right lateral incisor was extracted from three individuals during the Late Jomon period. These individuals include one whose maxillary and mandibular lateral incisors were extracted in Phase I and another whose maxillary lateral incisor and canine were extracted in Phase II. Extraction patterns of the maxillary lateral incisor have been observed in the Hokkaido, Honshu, and Kyushu regions during the end of the Middle Jomon to the middle of the Late Jomon period (Harunari, 2002). These patterns showed the same tendency as that observed during the Late Jomon period. The type 2C individual was found in Phase II. The end of the Late Jomon period would have been the starting point of type 4I and 2C extractions. Three type 4I2C individuals were found in Phases II and III. This type of tooth extraction was widely observed during the Final Jomon period on the Atsumi Peninsula (Harunari, 1979, 2002). One type 4I2C individual belongs to the Final Jomon period and this is an ordinary case. The finding of these type 4I2C individuals during the Initial to Middle Yayoi period suggests a degree of cultural continuity from the Jomon period. Overall, the tooth extraction patterns of individuals from the Kawaji shell mound represent a rare case that shows the transition in tooth extraction patterns within a single site.

**Figure 6.**
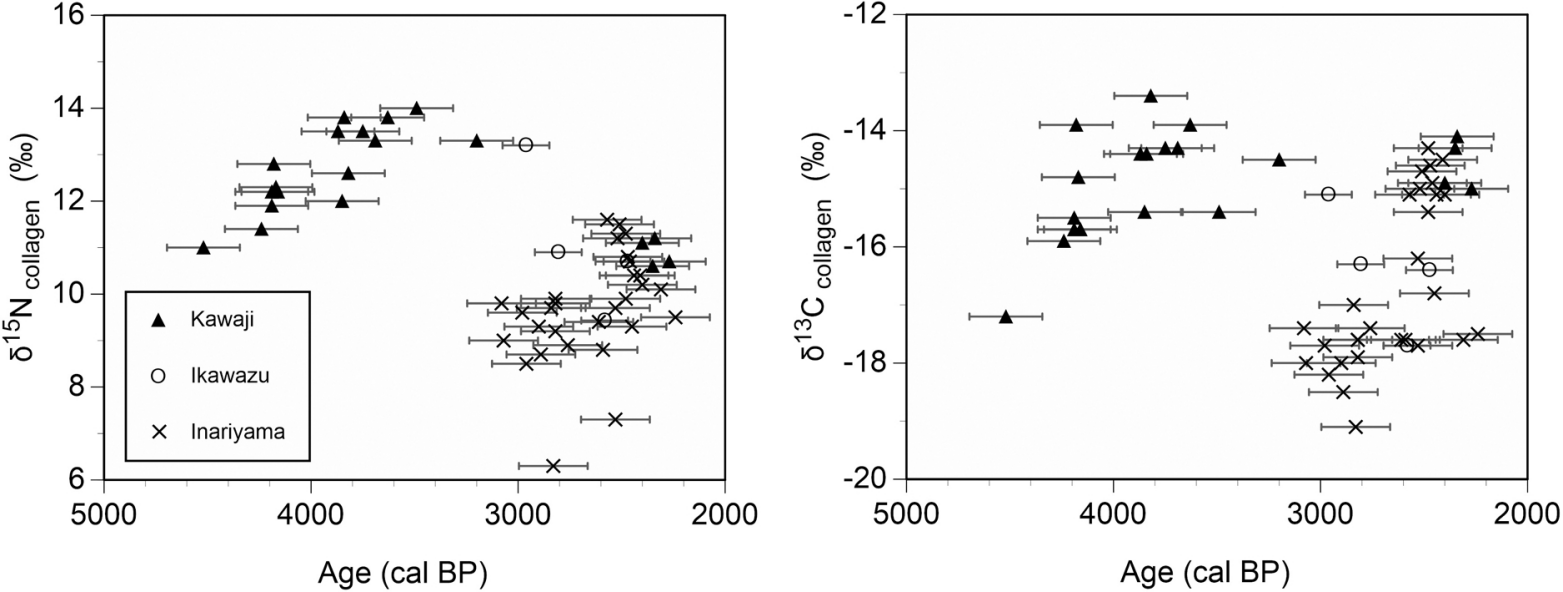
The δ^13^C and δ^15^N values and radiocarbon dates of human skeletal remains from the Kawaji, Ikawazu, and Inariyama shell mounds. Half the average of the 2σ range of calibrated dates was ±176 years for Kawaji, ±113 years for Ikawazu, and ±166 years for Inariyama (error bars).

The dates and δ^13^C and δ^15^N values of the human skeletal remains excavated from the Kawaji shell mound were compared with those reported for the Ikawazu shell mound, which is also located in the middle of the Atsumi Peninsula, and those of the Inariyama shell mound on the Toyokawa Plain (Figure 6; Kusaka et al., 2015, 2018). The dates of Ikawazu and Inariyama individuals were then plotted from the end of the Late Jomon to the Middle Yayoi periods. The δ^13^C and δ^15^N values for the Inariyama individuals during around 3200–2800 calBP tended to be lower than those of the other sites. The Kawaji individuals of the Initial to Middle Yayoi period exhibited δ^13^C and δ^15^N values as high as those of Inariyama individuals. Both Kawaji and Inariyama individuals, who exhibit equivalent isotopic values, would have incorporated similar proportions of marine resources into their diets during that time.

The locations of burial sites in the Kawaji shell mound were separated into two areas during Phases I and II (Figure 5). Most individuals were buried in the western part, while only two in Phases I and II were buried in the eastern part. Individuals in Phase III were buried in a row. The locations in the western and eastern parts are referred to as areas A and B, respectively. Most Phase I individuals were buried in the north and center parts of area A, while most Phase II individuals were buried in the southwestern part of area A. The concentrated burial sites in a cemetery were recognized as burial subgroups, which were made up of kin-related groups (Yamada, 2022b). The uneven distribution of burial sites in area A indicates that burial subgroups existed in Phase I and II and moved slightly to the south from Phase I to Phase II. One burial subgroup of Phase III appears in area B. Radiocarbon dating of human skeletal remains also revealed burial subgroups in the Kawaji shell mound.

The age-related burial subgroups and their dietary relationships are also interesting. The changes in δ^13^C and δ^15^N values over time may indicate changes in diet and subsistence. There are two possible interpretations regarding the use of the shell mound. One is that a kin-related group of Kawaji left the burial sites over time, and the other is that three different kin-related groups used the shell mound in different time periods. Cranium and tooth size measurements suggested similar intra- and inter-site morphological variation, which may indicate high consanguinity in regional Jomon populations (Kondo, 1994; Kondo et al., 2022). A comparison of pilasteric indexes between the unique burials of the *banjo-shuseki-bo* and the individual burials of Hobi suggested the possibility that people who engaged in physically demanding labor or were in a kin-related group might have been buried in the *banjo-shuseki-bo* (Mizushima et al., 2022). Strontium isotope analysis indicated people who migrated between sites were buried in Yoshigo and Inariyama, and immigrants would be included in the *banjo-shuseki-bo* (Kusaka et al., 2011, 2022). A kinship analysis based on ancient DNA has been attempted in a burial subgroup of Ikawazu (Waku et al., 2022). Although gradual changes in diet and tooth ablation patterns of Kawaji individuals were observed, the exact causes of these changes remain unclear and await further analyses.

## 5. Summary

Carbon and nitrogen isotope analysis and radiocarbon dating of human skeletal remains from the Kawaji shell mound were conducted to evaluate changes in diet over time. The radiocarbon dates indicated the end of the Middle to Final Jomon and Initial to Middle Yayoi periods. The higher δ^15^N values for individuals during the second half of the Late Jomon period indicated the presence of active fishing along the coast and in the open sea, whereas the lower δ^15^N values of individuals during the Initial to Middle Yayoi period indicated a decreased dependence on marine resources. The ritual tooth ablation patterns changed from the extraction of maxilla lateral incisors to types 2C and 4I2C. The locations of the burial sites were unevenly distributed in the shell mound in each phase. These findings regarding changes in the diet, types of tooth ablation, and burial locations over time from human skeletal remains excavated from the Kawaji shell mound are therefore an interesting case that warrants further study in the future.

## Figures and Tables

**Table 1.** Chronological division of the Jomon and Yayoi periods

Period		Age (calBP)	Reference
Jomon	Incipient	15540–11345	Kobayashi, 2017
	Initial	11345–7050	Kobayashi, 2017
	Early	7050–5415	Kobayashi, 2017
	Middle	5415–4490	Kobayashi, 2017
	Late	4490–3220	Kobayashi, 2017
	Final (first half of Final Jomon)	3220–2850	Yamamoto, 2008; Shitara and Kobayashi, 2004
Jomon/Yayoi	Initial Yayoi (second half of Final Jomon)	2850–2450?	Yamamoto, 2008; Shitara and Kobayashi, 2004
Yayoi	Early	2460–2305	Kobayashi, 2017; Shitara and Kobayashi, 2004
	Middle	c. 2350–2100	Fujio and Ozaki, 2009

**Table 2.** Results of stable isotope analysis and radiocarbon dating on human skeletal remains from the Kawaji shell mound

Excavation No.	Kiyono Collection No.	Sex	Age at death	Ritual tooth ablation	Col%	C%	N%	C/N	δ^13^C (‰)	δ^15^N (‰)	Marine%	Lab. Code	Radiocarbon dates			Calibrated dates (cal BP)		Phase	Period	Reference
													(BP)		(1SD)	(2σ, from, to)	(Median)			
8	172	UNK	YAd	No RTA	1.5	40.7	13.4	3.5	–17.2	11.0	31.8	PLD-35532	4170	±	25	4800–4390	4520	I	Middle/Late Jomon	Kusaka et al., 2010
9	173	UNK	MAd	No RTA	0.7	42.3	14.8	3.3	–15.9	11.4	42.8	PLD-36625	4000	±	20	4410–4090	4240	I	Late Jomon	Kusaka et al., 2010
11	175	M	Adult	UNK	0.9	42.6	15.0	3.3	–15.5	12.2	47.2	PLD-35533	3980	±	25	4390–4000	4190	I	Late Jomon	Kusaka et al., 2010
22	186	M	YAd	UNK	1.0	38.0	12.8	3.5	–15.7	11.9	45.1	PLD-35539	3970	±	25	4380–4000	4190	I	Late Jomon	This study
13	177	M	YAd	RI^2^	2.6	42.5	15.0	3.3	–13.9	12.8	61.5	PLD-33237	4030	±	20	4360–3980	4180	I	Late Jomon	This study
17	181	F	MAd	RI^2^andLI_2_	1.4	41.1	14.3	3.3	–14.8	12.3	53.4	PLD-33238	3990	±	20	4360–3980	4170	I	Late Jomon	Kusaka et al., 2010
23	187	UNK	Adult	UNK	2.2	42.9	14.9	3.4	–15.7	12.2	45.0	PLD-36628	3950	±	20	4350–3970	4160	I	Late Jomon	This study
18	182	M	MAd	No RTA	1.5	41.7	14.7	3.3	–14.4	13.5	56.8	PLD-33239	3780	±	20	4070–3690	3870	II	Late Jomon	Kusaka et al., 2010
19	183	UNK	Adult	UNK	1.8	42.1	14.8	3.3	–15.4	12.0	47.7	PLD-35537	3740	±	25	3990–3690	3850	II	Late Jomon	This study
10	174	M	MAd	No RTA	1.0	42.8	14.9	3.3	–14.4	13.8	56.7	PLD-33236	3760	±	20	3980–3650	3840	II	Late Jomon	Kusaka et al., 2010
21	185	M	MAd	RI^2^C	1.5	42.9	14.8	3.4	–13.4	12.6	66.2	PLD-35538	3780	±	20	3980–3640	3820	II	Late Jomon	This study
15	179	F	YAd	2C	0.9	41.3	14.1	3.4	–14.3	13.5	58.0	PLD-35535	3690	±	25	3890–3570	3750	II	Late Jomon	Kusaka et al., 2010
14	178	UNK	YAd	No RTA	2.6	43.8	15.2	3.4	–14.3	13.3	57.7	PLD-36626	3650	±	20	3840–3530	3690	II	Late Jomon	This study
16	180	M	YAd	UNK	0.9	39.9	13.0	3.6	–13.9	13.8	60.9	PLD-35536	3620	±	20	3820–3460	3630	II	Late Jomon	Kusaka et al., 2010
12	176	UNK	Adult	UNK	1.3	43.1	14.0	3.6	–15.4	14.0	47.9	PLD-35534	3460	±	20	3630–3370	3490	II	Late Jomon	This study
7	171	M	Adult	4I2C	1.0	42.2	13.5	3.6	–14.5	13.3	55.9	PLD-36624	3220	±	20	3360–3020	3200	II	Late/Final Jomon	This study
5	169	UNK	Adult	UNK	1.3	41.9	13.7	3.6	–14.9	11.1	52.3	PLD-36623	2570	±	20	2680–2200	2400	III	Initial/Early/Middle Yayoi	This study
2	166	F	MAd	4I2C	2.1	42.3	14.8	3.3	–14.3	10.6	57.8	PLD-33234	2550	±	20	2490–2160	2350	III	Initial/Early/Middle Yayoi	Kusaka et al., 2010
6	170	UNK	Adult	No RTA	1.9	41.0	13.9	3.4	–14.1	11.2	59.2	PLD-33235	2550	±	20	2490–2160	2340	III	Initial/Early/Middle Yayoi	This study
1	165	F	MAd	4I2C	3.2	40.5	13.6	3.5	–15.0	10.7	51.1	PLD-33233	2470	±	20	2410–2110	2270	III	Early/Middle Yayoi	This study
4	168	UNK	Adult	UNK	1.2	42.7	13.6	3.7	–15.0	11.7	—	PLD-36622	2500	±	20	—	—	—		This study
20	184	UNK	Adult	UNK	0.6	39.7	11.9	3.9	–16.0	12.5	—	PLD-36627	3770	±	20	—	—	—		This study

M, male; F, female; UNK, unknown; YAd, young adult; MAd, middle adult; No RTA, no ritual tooth ablation; UNK, ritual tooth ablation is unknown.

**Table 3.** Summary statistics of stable isotope analysis on human skeletal remains from the Kawaji shell mound

Category	N	δ^13^C (‰)			δ^15^N (‰)	
		Mean	SD		Mean	SD
All	20	–14.9	0.9		12.4	1.1
Phase I	7	–15.5	1.0		12.0	0.6
Phase II	9	–14.4	0.6		13.3	0.6
Phase III	4	–14.6	0.4		10.9	0.3
